# Overexpression of *PpSnRK1α* in Tomato Promotes Fruit Ripening by Enhancing RIPENING INHIBITOR Regulation Pathway

**DOI:** 10.3389/fpls.2018.01856

**Published:** 2018-12-17

**Authors:** Wen Yu, Futian Peng, Yuansong Xiao, Guifang Wang, Jingjing Luo

**Affiliations:** ^1^State Key Laboratory of Crop Biology, College of Horticulture Science and Engineering, Shandong Agricultural University, Tai’an, China; ^2^Shandong Institute of Pomology, Tai’an, China

**Keywords:** SnRK1 protein kinase, RIN, fruit ripening, peach, tomato

## Abstract

As a conserved kinase complex, sucrose non-fermenting-1-related protein kinase 1 (SnRK1) is a major regulator of plant growth and development. In our previous study, overexpression of *MhSnRK1* in tomato (*Solanum lycopersicum* L.) modified fruit maturation: the transgenic fruit ripened earlier than the wild type (WT). However, the mechanism by which fruit maturation is regulated by SnRK1 is not clear; therefore, the test materials used were the transgenic tomato lines (OE-1, OE-3, and OE-4) overexpressing the coding gene of peach [*Prunus persica* (L.) Batsch] SNF1-related kinase α subunit (*PpSnRK1α*). The activity of SnRK1 kinase in transgenic tomato lines OE-1, OE-3, and OE-4 was higher than that in the WT at different periods of fruit development; in the pink coloring period the SnRK1 kinase activity increased the most, with 23.5, 28.8, and 21.4% increases, respectively. The content of starch and soluble sugars in red ripe transgenic fruit significantly increased, while the soluble protein and titratable acid content decreased significantly. We also found that the tomatoes overexpressing *PpSnRK1α* matured approximately 10 days earlier than the WT. Moreover, the yeast-two-hybrid assay showed that PpSnRK1α interacted with the MADS-box transcription factor (TF) SIRIN, which acts as an essential regulator of tomato fruit ripening. The BiFC technology further validated the location of the PpSnRK1α interaction sites within the nucleus. The quantitative real-time PCR analysis showed that *RIN* expression was up-regulated by *PpSnRK1α* overexpression; the expression of RIN-targeted TF genes *NOR* and *FUL1* increased during different stages of fruit development. The expression of key genes, *ACS2*, *ACS4*, and *E8*, in ethylene synthesis also changed accordingly, and the ethylene emitted by the red ripe fruit increased by 36.1–43.9% compared with the WT. These results suggest that PpSnRK1α interacts with SIRIN, increasing the expression of *RIN*, thereby regulating the expression of downstream ripening-related genes, finally promoting fruit ripening.

## Introduction

Sucrose non-fermenting 1 kinase (SNF1)-related kinase (SnRK1) in plants belongs to a conserved family that includes SNF1 in yeast and AMP-activated protein kinase in animals (Crozet et al., 2014). SnRK1 is a heterotrimeric protein complex that is an important kinase in the signal transduction of carbon and nitrogen and is one of the regulatory hubs in plant physiological activities (Le Guen et al., 1992; Halford and Hardie, 1998; Polge and Thomas, 2007). Previous studies have shown that SnRK1 may play a key role in the overall regulation of the intracellular sugar signaling pathway and metabolism, and regulates plant carbohydrate metabolism (Ramon et al., 2013; Emanuelle et al., 2016). Recently, many studies have shown that plant SnRK1 is involved in many metabolic pathways including carbohydrate metabolism, stress, organogenesis, and senescence pathways (Purcell et al., 1998; Laurie et al., 2003; Jossier et al., 2009; Broeckx et al., 2016).

Most research on the function of SnRK1 has been conducted in *Arabidopsis thaliana* and crop plants, while research on the function of SnRK1 in fruit trees has rarely been reported. Our previous study showed that overexpression of Pingyitiancha (*Malus hupehensis* Rehd. var. *pingyiensis* Jiang) *MhSnRK1* in tomato can improve the photosynthetic rate, fruit soluble sugar content, starch content and utilization, and also influence the process of growth and development of fruit—for example, transgenic tomato fruit matured 10 days earlier than WT fruit (Li et al., 2010; Wang et al., 2012). This study was also the first to show that SnRK1 affects fruit ripening (Wang et al., 2012).

Tomato is a climacteric fruit, and early studies of the molecular genetic mechanism during the maturation process focused on signal transduction of ethylene biosynthesis and ethylene receptor mediated regulation. With a deep understanding of the ethylene pathway, researchers have gradually realized that if a fruit only has ethylene, it is not mature. Only up to a certain stage of development is a fruit sensitive to ethylene stimulation (Wilkinson et al., 1995). Therefore, the problem of upstream regulation of the ethylene pathway has become a new research focus. As typical of many mutants, the *rin* mutation exists in the upper reaches of the ethylene regulatory pathway, and this is not regulated by ethylene. The MADS-box TF RIN was cloned in the tomato *rin* gene locus using map-based cloning described by Vrebalov et al. (2002), and a homologous gene was also found in strawberry (a model plant for studying the non-respiratory climacteric pathway). Thus, it was inferred that RIN may be a conserved regulatory factor for two types of fruit (Vrebalov et al., 2002). Previous research has shown that RIN is a member of the MADS-box gene family and is a very important factor in the regulation of tomato fruit ripening, affecting almost all relevant metabolic pathways (Vrebalov et al., 2002; Martel et al., 2011). The RIN protein can not only directly regulate fruit ripening-related genes, such as lipid and cell wall metabolism genes, but also can influence ethylene synthesis pathways and other ripening-related TFs (e.g., CNR, NOR, FUL1, and AP2a), indicating that RIN has a very important function in tomato fruit ripening (Fujisawa et al., 2011, 2013).

Our previous study found that the overexpression of *MhSnRK1* in tomato can promote fruit ripening (Wang et al., 2012); however, the exact molecular mechanism by which SnRK1 regulates fruit maturation is not clear. Does SnRK1 interact with the TF RIN, regulating fruit ripening? Using *PpSnRK1α* overexpressing tomato lines (OE-1, OE-3, and OE-4) and WT tomato as test material, we examined the relationship between SnRK1 and RIN and we speculate that SnRK1 regulates fruit maturation by affecting the RIN regulation pathway.

## Materials and Methods

### Plant Material and Treatments

We previously obtained the transgenic lines OE-1, OE-3, and OE-4 overexpressing *PpSnRK1α*. Seeds of transgenic and WT tomatoes (*Solanum lycopersicum ‘*Sy12f*’*) were germinated and grown in a plant growth chamber at 30°C for 3 weeks. These transgenic tomatoes (T2) were confirmed using the Plant PCR Kit (Takara, Japan). The primers *PpSnRK1α*-F (5′-GCTCTAGAATGGATGGATCGGTTG-3′) and *PpSnRK1α*-R (5′-GCGTCGACTTAAAGGACCCG-3′) were used to detect *PpSnRK1α* overexpressing tomato plants. The PCR-positive tomato plants were transplanted into pots with soil, and both WT and transgenic tomato plants were grown under natural light. Fifteen tomato plants per genotype were used (one plot per five plants, three plots per line treatment). All tomato fruits of the WT and transgenic plants used for analysis were tagged at the date of anthesis, and the fruit ripening time was observed. For fruit diameter and ripening time analysis, fifteen fruit samples per plot were used and three independent replicates were performed. The WT and transgenic fruits were harvested at different ripening stages, viz., green mature (GM), breaker (BK), pink coloring (PK), and red ripe (RR) stage, to analyze the SnRK1 activity, the gene expression level and ethylene emission as well as the soluble sugar, starch, soluble protein, and titratable acid content. In each case, three biological replicates were performed and each replicate contained at least 10 fruits.

### Quantitative Real-Time PCR

Total RNA was extracted from the tomato fruits at different stages of development using the RNA plant Plus Reagent kit (TIANGEN, China). The RNA was then reverse-transcribed to cDNA using a Primescript^TM^ RT reagent kit (Takara, Japan). qRT-PCR was performed using SYBR Premix Ex Taq^TM^ (Takara, Japan). The *r18S* gene was used as loading controls. The calculation method for qRT-PCR is 2^-ΔΔCT^. Three independent biological replicates were analyzed per sample. The specific primers used for the PCR analysis are listed in Table 1.

**Table 1:** Primers used in this study.

Gene	Acc. no	Primer sequences (5′–3′)
*SIRIN*	AF448522	F: CATGGCATTGTGGTGAGCAAAGTGT R: AGCATCATGTGTTGATGGTGCTGC
*SINOR*	AY573802.1	F: AGAGAACGATGCATGGAGGTTTGT R: ACTGGCTCAGGAAATTGGCAATGG
*SIFULI*	X60757.1	F: ACTGGACTCTCCTCACCTTGGGG R: AGCTGCACCTTGCTGCTGTGA
*SIACS2*	X59139	F: AAGCGCGATGAGGTTAGGTA R: AAAGTGGACGCAAATCCATC
*SIACS4*	M88487	F: AAATCTCCACCTTCACTAACGAAC R: CCTAAGTCCTTGGAAAGACTAGACAC
*SIE8* *SISNF1* *SIr18S*	DQ317599 AF143743 X51576	F: TGGCTCCGAATCCTCCCAGTCT R: GTCCGCCTCTGCCACTGAGC F: CGCAGATTTTGGTTTGAGCAA R: GTTTGGGCTTCCGCAACTT F: GCCCGGGTAATCTTTGAAAT R: AGTAAGCGCGAGTCATCAGC
*PpSnRK1*α *PpSnRK1*α (JM) *SIRIN* (JM)	ppa004347m ppa004347m AF448522	F: CTCTTG GTATTGGTTCTT R: TCTCTTCTCACTTTCTCT F: GAATTCATGGATGGATCGGTTGGC R: GTCGACTTAAAGGACCCGAAGTTGT F: TCCCCCGGGGTACAATATGGGTAGAGGGAAAG R: AAACTGCAGTCAAAGCATCCATCCAGGT


### SnRK1 Activity Assays

Fruit tissue (1 g) was ground in 1 mL of cold extraction buffer consisting of 100 mmol⋅l^-1^ HEPES, pH 8, 25 mmol⋅l^-1^ NaF, 2 mmol⋅l^-1^ sodium pyrophosphate, 0.5 mmol⋅l^-1^ethylene diamine tetra acetic acid, 0.5 mmol⋅l^-1^ ethylene glycol tetra acetic acid, 1 mmol⋅l^-1^ anisole, 5 mmol⋅l^-1^ dithiothreitol, 25 mmol⋅l^-1^ β-mercaptoethanol, and 1 mol⋅l^-1^ pepstatin A. The suspension was transferred to two cold microfuge tubes and clarified by centrifugation for 5 min at 12,000 ×*g* at 4°C. The supernatant (750 μL) was desalted on a 2.5 mL centrifuge column (Sephadex G-25 medium columns; GE Healthcare, United Kingdom) treated with equilibration solution. Using AMARA polypeptide as the substrate (Zhang et al., 2009), the SnRKl activity was measured using a Universal Kinase Activity Kit (R&D Systems, Minneapolis, MN, United States).

### Yeast Two-Hybrid Assay

For the yeast two-hybrid experiments, the plasmids pGAD424 and pGBT9 were used, which contain the GAL4 activation domain and GAL4 DNA-binding domain, respectively. *SIRIN* was amplified and then cloned into the pGBT9 vector. *PpSnRK1α* was amplified and then inserted into pGAD424. The BT-RIN and AD-SnRK1α plasmids were co-transformed into the yeast strain Y2HGold (Clontech, Palo Alto, CA, United States) using the PEG/LiAC method as described in the Clontech Yeast Protocol Handbook. The transformed colonies were selected on synthetic drop-out medium lacking leucine and tryptophan (SD-Leu-Trp). The colonies from the double selection plates were then screened for growth on quadruple selection SD medium lacking adenine, histidine, leucine, and tryptophan (SD-Ade-His-Leu-Trp). To further confirm the positive interactions, X-alpha-Gal was used to assay for beta-galactosidase activity. Primers are listed in Table 1.

### Bimolecular Fluorescence Complementation (BiFC) Assay

Full-length *PpSnRK1α* and *SIRIN* were transferred from their respective entry clones into the vectors pSPYNE and pSPYCE. Plasmids were co-transfected into Agrobacterium. Following the methods described by Boruc et al. (2010), the instantaneous expression of Agrobacterium-mediated tobacco leaves was used to detect protein interactions, and the fluorescence results were observed with a confocal laser scanning microscope (Zeiss 510 Meta).

### Analyses of Soluble Sugar and Starch in Fruit

Soluble sugar was extracted from 1 g of each sample, which was placed in 10 mL of water at 100°C, and then extracted twice at 100°C with the same volume of water (Wang et al., 2012). The total amount of soluble sugar was determined using the anthrone method (van Herwaarden et al., 1998). Starch was determined in the remaining sample after the soluble sugars were extracted. The tissue residue was digested with 0.92 mol⋅L^-1^ perchloric acid in 20 mL water at 100°C for 30 min to convert starch to glucose. This digestion was repeated twice. The amount of glucose was then determined using the anthrone method.

### Measurements of Titratable Acid and Soluble Proteins

Following the methods presented by Wang et al. (2012), titratable acid content of isolated juice sacs was determined by titration. Fresh tissue (50 g) was ground completely using a mortar and pestle and placed in 5 mL of 80% ethanol at 80°C for 1 h. Aliquots of the ethanol extracts were titrated to a neutral endpoint with 0.1 mol⋅L^-1^ sodium hydroxide, indicated by phenolphthalein. Proteins were extracted from 0.5 g of tissue sample with 5 mL of enzyme assay buffer (5 mmol⋅L^-1^ cysteine, 5 mmol⋅L^-1^ EDTA-Na_2_, and 25 mmol⋅L^-1^ potassium phosphate buffer at pH 7.5), ground with a mortar and pestle, and centrifuged (4,000 r/min) for 15 min. The protein content of the supernatant was measured at 500 nm following the method described by Lowry et al. (1951) using bovine serum albumin as the standard protein.

### Determination of Ethylene Emittance

The determination of ethylene emitted by tomato fruit referenced the method described by Jin et al. (2006). The Shimadzu GC-9A gas chromatograph was used with N_2_ as the carrier, and the separation column and detector temperatures were 40°C and 120°C, respectively. Samples were taken from three glass containers and the ethylene emitted was calculated according to the peak area method.

### Statistical Analysis

Three independent biological replicates were performed for each experiment. The statistical analysis was performed with Microsoft Office Excel 2007 software. Comparison of the means was calculated according to the Duncan multiple range test using the SPSS 20.0 statistical program. Significance was defined as *P <* 0.05.

## Results

### *PpSnRK1α* Transgenic Fruit Ripens 10 days Earlier Than WT Fruit

**FIGURE 1:**
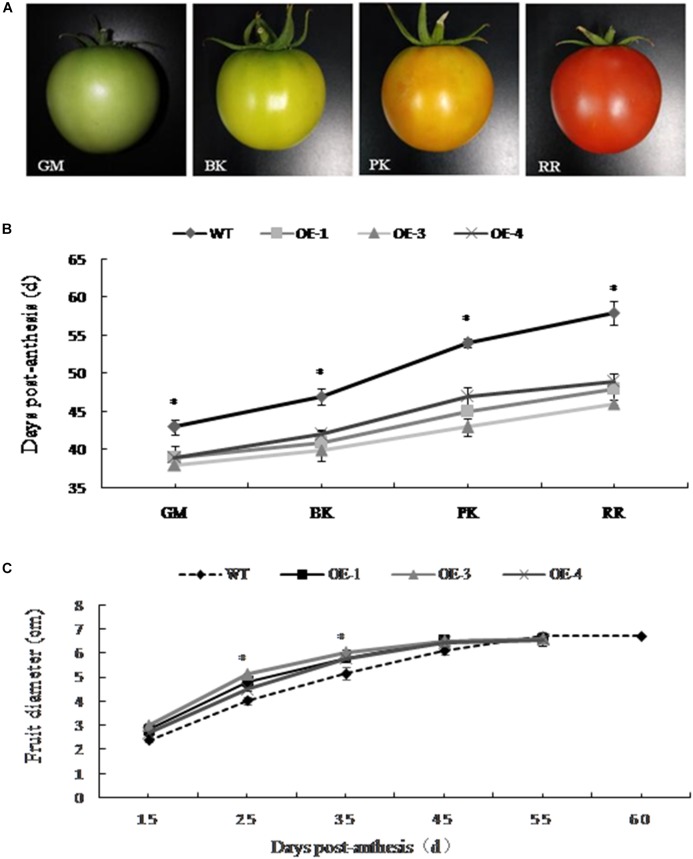
Effect of *PpSnRK1α* overexpression on transgenic tomato fruit development. **(A)** Changes in the appearance of fruit color on which the four developmental stages were divided. Tomato fruit developmental stages: green mature (GM); breaker (BK); pink coloring (PK); red ripe (RR). **(B)** Days required for the different fruit developmental stages; **(C)** Changes in fruit diameter in transgenic and wild type (WT) tomato plants. Error bars represent the SD based on three independent biological replicates. An asterisk (^∗^) on top of the error bar designates a significant difference between transgenic lines and WT at *P* < 0.05.

The development of *PpSnRK1*α transgenic tomato plants (OE-1, OE-3, and OE-4) and WT fruit was observed in real time. The changes of fruit diameter and color and the number of days required for fruit development to reach different stages were recorded (Figures 1A–C). According to the changes in the appearance of fruit color, four different developmental stages were divided (Figure 1A). The development of *PpSnRK1α* overexpressing tomatoes was significantly faster than that of WT tomatoes, and the fruit matured approximately 10 days earlier than WT fruit (Figure 1B). In the early stage of fruit development, the diameter of the transgenic tomato fruit was significantly larger than that of the WT, but there was no significant difference in fruit size between the two since the green mature stage (Figure 1C).

### Higher Starch and Soluble Sugar Content in *PpSnRK1α* Transgenic Tomato Than WT

The soluble sugar content in red ripe fruit from the OE-1, OE-3, and OE-4 tomato lines was significantly higher than that of the WT, increasing by 33.9, 38.4, and 32.5%, respectively (Table 2). The starch content in the fruit also significantly increased, and the starch content in the OE-3 fruit was nearly twice that of the WT. The soluble protein and titratable acid content in fruit was lower than that of the WT. The overexpression of *PpSnRK1α* affects the accumulation and distribution of carbohydrates in tomato fruit.

**Table 2:** The starch, soluble sugar, soluble protein, and titratable acid content in WT and transgenic tomato fruit.

	Starch (mg⋅g^-1^ FW)	Soluble sugar (mg⋅g^-1^ FW)	Soluble protein (mg⋅g^-1^ FW)	Titratable acid (% FW)
WT	4.23 ± 0.28 c	26.62 ± 1.38 b	0.51 ± 0.04 a	0.40 ± 0.03 a
OE-1	7.32 ± 0.36 b	35.64 ± 0.40 a	0.34 ± 0.05 b	0.32 ± 0.01 b
OE-3	8.26 ± 0.45 a	36.83 ± 0.70 a	0.36 ± 0.03 b	0.33 ± 0.04 b
OE-4	7.13 ± 0.77 b	35.27 ± 0.43 a	0.34 ± 0.03 b	0.34 ± 0.02 b


*Data in the table are the average of three samples; lowercase letters indicate a significant difference at *P* < 0.05.*

### Expression Analysis of SnRK1 Gene and Activities of SnRK1 in Transgenic and WT Tomato

**FIGURE 2:**
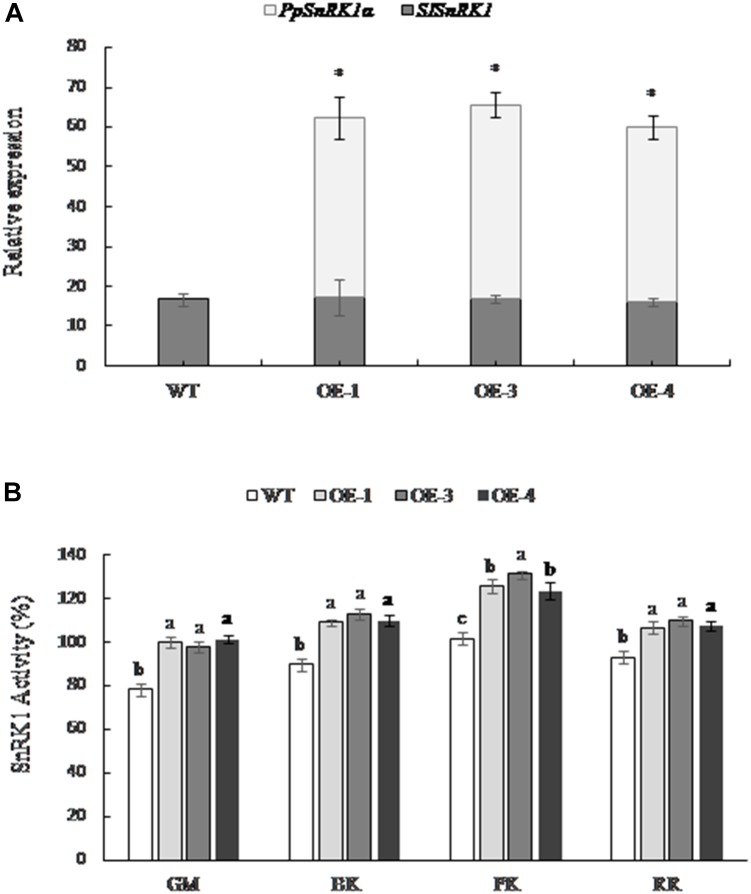
The expression of *SnRK1α* and the activity of SnRK1 in fruit from *PpSnRK1α* overexpressing tomato lines (OE-1, OE-3, and OE-4) and the WT during different developmental stages. **(A)** The expression of the *PpSnRK1α* gene and *SISNF1* gene in tomato fruit. The error bars represent the SD of three biological replicates. An asterisk (^∗^) on top of the error bar designates a significant difference between transgenic lines and the WT at *P* < 0.05. **(B)** The SnRK1 kinase activity in tomato fruit at different developmental stages. The error bars represent the SD of three biological replicates. Different lowercase letters in the same developmental stage indicate a significant difference at *P* < 0.05.

The expression of the tomato SnRK1 encoding gene (*SISNF1*) in transgenic tomatoes OE-1, OE-3, and OE-4 was consistent with the WT fruit; however, the expression of *PpSnRK1α* was higher in transgenic tomato lines, and no expression was detected in the WT (Figure 2A), indicating that *PpSnRK1α* was successfully expressed in transgenic tomato lines and did not affect the expression of *SISNF1* in tomato. The SnRK1 activity was significantly higher in OE-1, OE-3, and OE-4 fruit than that of WT tomatoes at different periods, and the SnRK1 activity showed the greatest increase at the pink coloring stage, increasing by 23.5, 28.8, and 21.4%, respectively (Figure 2B).

### SnRK1–RIN Interaction Identified by the Yeast Two-Hybrid System

**FIGURE 3:**
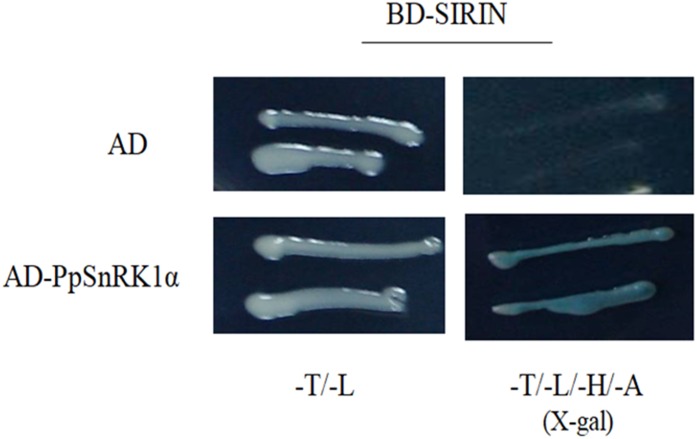
Yeast two-hybrid experiments showing the interaction between PpSnRK1α and SIRIN protein. Results show a representative experiment out of three independent biological replicates.

We performed yeast two-hybrid (Y2H) assays to determine whether PpSnRK1α interacts with SIRIN. For Y2H assays, the full-length *SIRIN* was inserted into a pGBT9 vector as bait. The results indicated that RIN protein did not show auto-activation (Figure 3). The bait construct carrying the BD-RIN fusion protein was co-transformed with the prey construct harboring the AD-SnRK1α fusion protein, indicating that PpSnRK1α interacted with SIRIN (Figure 3).

### SnRK1–RIN Interaction Identified by BiFC

**FIGURE 4:**
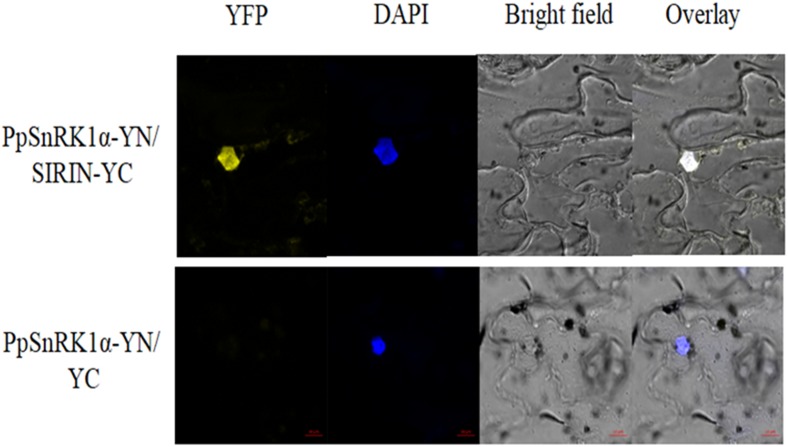
Analysis of interaction between PpSnRK1α and SIRIN by BiFC assay. Visualization of the protein complex using BiFC in tobacco leaf epidermal cells. Results show a representative experiment out of three independent biological replicates.

On the basis of the Y2H experiment, this study used a bimolecular fluorescence complementary (BiFC) test to further prove the interaction between PpSnRK1 and the TF SIRIN *in vivo*. As shown in Figure 4, tobacco leaves were co-infected with Agrobacterium containing PpSnRK1α-pSPYNE and SIRIN-pSPYCE recombinant plasmids, and yellow fluorescence (YFP) signal was observed in the epidermal nucleus of tobacco leaves. The positions of YFP signal and blue fluorescence signal were exactly the same. The results showed that PpSnRK1α protein could interact with SIRIN protein in plants, and their interaction sites were in the nucleus.

### Expression of RIN at Different Developmental Stages of Tomato Fruit Overexpressing *PpSnRK1α*

**FIGURE 5:**
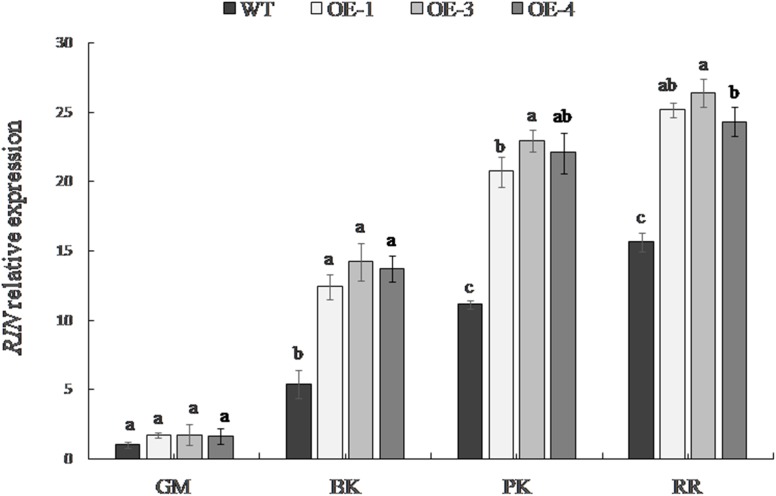
*RIN* expression in WT and the *PpSnRK1α* overexpressing tomato fruit. Averages of three biological replicates ± SD are shown. Different lowercase letters in the same developmental stage indicate a significant difference at *P* < 0.05.

The expression level of *RIN* differed significantly among the fruit developmental stages (Figure 5). The *RIN* gene was expressed at a low level in the green mature stage; its expression gradually increased with the maturity of the fruit and was the greatest in the red ripe stage. The expression of the *RIN* gene in transgenic fruit was significantly higher than that in WT tomato from the breaker stage. The up-regulation of *RIN* expression was most significant at the pink coloring stage in *OE-1, OE-3*, and *OE-4*, which was up to 1.86, 2.06, and 1.98 times the *RIN* expression in the WT, respectively.

### In *PpSnRK1α* Overexpressing Fruit, RIN Regulates Gene Expression in the Maturity Pathway

**FIGURE 6:**
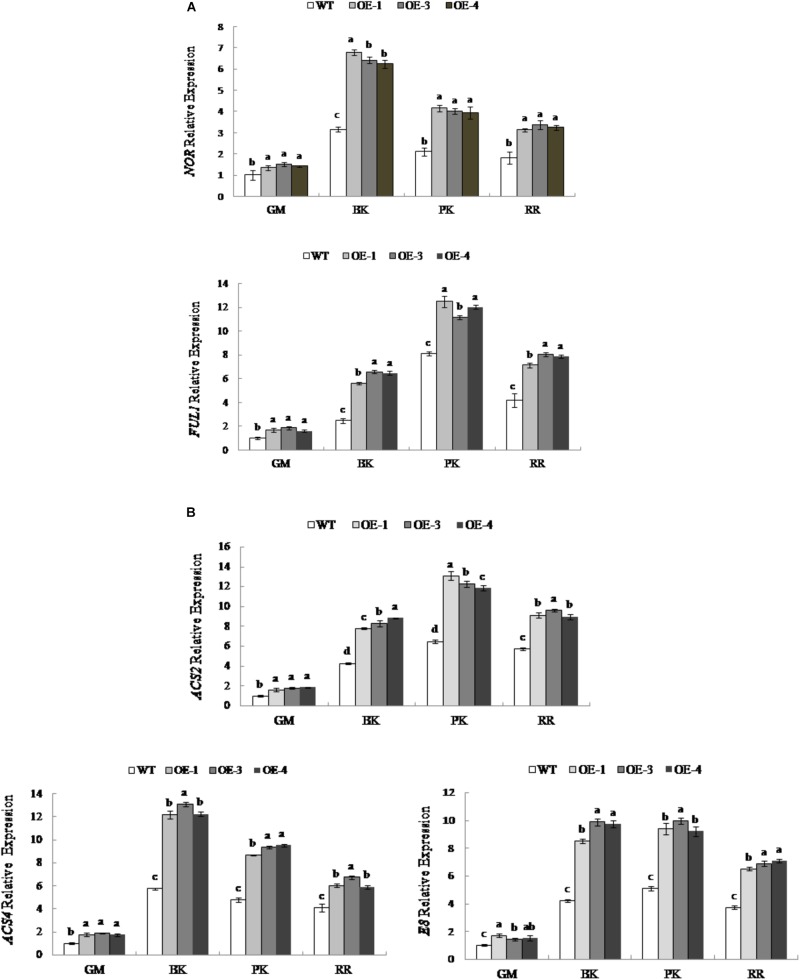
Expression of RIN-targeted genes in WT and *PpSnRK1α* overexpressing tomato fruit during different fruit developmental stages. **(A)** ripening-related transcription factors; **(B)** ethylene synthesis enzymes. Error bars represent the SD based on three independent biological replicates. Different lowercase letters in the same developmental stage indicate a significant difference at *P* < 0.05 level.

To explore the effects of changes in *RIN* expression on fruit ripening, the expression levels of RIN-targeted genes associated with maturation were examined. We select RIN-targeted TF genes *NOR* and *FUL1* and key genes for ethylene synthesis, *ACS2*, *ACS4*, and *E8*, which are considered to be directly regulated by RIN (Eriksson et al., 2004; Ito et al., 2008; Fujisawa et al., 2011, 2013). As shown in Figure 6A, the expression levels of the TF *NOR* and *FUL1* genes were different during fruit development; the expression of *NOR* was the highest in the breaker period, while the expression of *FUL1* was the highest at the pink coloring stage. At different stages of fruit development, the expression levels of *NOR* and *FUL1* in *PpSnRK1α* overexpression tomato lines (OE-1, OE-3, and OE-4) were significantly higher than that of the WT. The expression was the most significantly up-regulated at the breaker stage in OE-1, OE-3, and OE-4 compared with that of the WT, and *NOR* was up-regulated by 1.15 times, 1.03 times, and 0.98 times, respectively, while *FUL1* increased by 1.24 times, 1.62 times, and 1.58 times, respectively.

The expression levels of the ACC synthase encoding genes *ACS2* and *ACS4* are different during fruit development. *ACS2* had the highest expression in the pink coloring stage, while *ACS4* had the highest expression in the breaker stage; the expression of the ACC oxidase encoding gene *E8* was similar to that of *ACS2* (Figure 6B). The expression levels of *ACS2*, *ACS4*, and *E8* in transgenic tomatoes OE-1, OE-3, and OE-4 were significantly higher than those in the WT at different stages of fruit development. In OE-1, OE-3, and OE-4, the expression of *ACS2* was the most up-regulated in the pink coloring stage, which, compared with the WT, increased by 2.01 times, 1.89 times, and 1.82 times, respectively. The expression of *ACS4* was up-regulated the most during the breaker stage in OE-1, OE-3, and OE-4, with values 2.12 times, 2.25 times and 2.13 times that of the WT, respectively. Similarly, *E8* was up-regulated the most during the breaker stage, with values in OE-1, OE-3, and OE-4 up to 2.02 times, 2.35 times, and 2.31 times that of the WT, respectively (Figure 6B). However, the genes not under the control of RIN were not up regulated (Supplementary Figure S1).

### Ethylene Emitted From Red Ripe Fruit of Transgenic and WT Tomato

**FIGURE 7:**
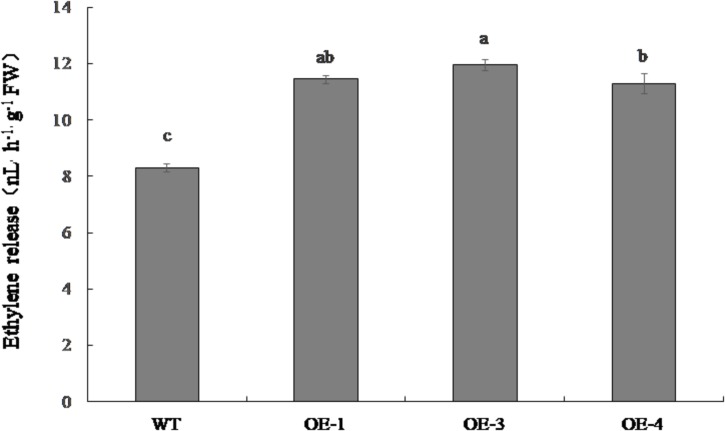
Ethylene release in red ripe fruit of *PpSnRK1α* overexpression tomato lines (OE-1, OE-3, and OE-4) and WT tomatoes. Error bars represent the SD based on three independent biological replicates. Different letters indicate statistically significant differences between the samples (*P* < 0.05).

The ethylene emitted from OE-1, OE-3, and OE-4 fruit was significantly higher than that of the WT, increasing by 37.9, 43.9, and 36.1%, respectively (Figure 7).

## Discussion

As a conserved energy sensor, SnRK1 plays an important role in plant metabolism, stress signal response, and plant growth and development (Hedbacker and Carlson, 2008; Carling et al., 2012). SnRK1 controls the early growth of pea cotyledons by coordinating metabolic, hormonal, and developmental signals that influence seed maturation (Radchuk et al., 2010). In our previous study, overexpression of *MhSnRK1* in tomato improved the photosynthetic rate, fruit soluble sugar content, starch content and utilization, and the transgenic tomato fruit matured 10 days earlier than the WT fruit (Li et al., 2010; Wang et al., 2012). In order to further explore the effect of SnRK1 on fruit maturation, transgenic tomatoes over expressing *PpSnRK1α* were studied. The results of the present study showed that overexpression of *PpSnRK1α* significantly increased the content of soluble sugar and starch in the fruit, and the fruit ripening period was 10 days earlier than WT fruit, which confirmed our previous results.

Hou (2009) performed a yeast two-hybrid system sieve library on the tomato TF RIN, finding that one of the proteins interacting with RIN is tomato LeSNF1/AMPK, which has phosphorylation activity. However, whether the combination of the two is just a coincidence of structural matching has not been further verified by the authors. In this study, the interaction between PpSnRK1α protein and tomato SIRIN protein was tested by a yeast two-hybrid experiment and BiFC assay. The results showed a positive interaction between SnRK1α and RIN. We also analyzed the expression of *RIN* at the transcriptional level and found that its expression level was significantly increased in *PpSnRK1α* overexpressing tomato, suggesting that SnRK1 regulated RIN at both the protein and transcriptional level.

RIN belongs to the family of typical MADS-box TFs, which are responsible for a series of physiological and biochemical processes such as respiration, photosynthesis, and nutrient metabolism. RIN are inhibited in *rin* mutants, and some genes related to fruit maturation are aberrantly expressed, indicating that RIN has a very important role in fruit ripening (Ng and Yanofsky, 2001; Vrebalov et al., 2002). Recently, researchers used ChIP, proteomics, gene chips, and other experimental techniques to show that the RIN protein directly targets genes involved in ethylene synthesis and signal transduction pathways, cell wall metabolism, and fruit softening; while in the tomato ripening process, many TF genes, such as *NOR*, *CNR*, *FUL1*, and *HB-1*, are also directly regulated by RIN (Fujisawa et al., 2013). The transcriptional levels of target genes related to maturation of RIN were also investigated in this study. The expression of *NOR* and *FUL1* TF genes in the tomato fruit breaker and pink coloring stages had corresponding increases; the expression levels of *ACS2*, *ACS4*, and *E8* in the ethylene pathway also increased with elevated *RIN* expression. FUL1 is a MADS-box TF with an expression pattern that suggests a possible role during tomato fruit ripening (Busi et al., 2003). NOR is a NAC-domain TF, and when mutated, shows a non-ripening phenotype similar to *rin* (Giovannoni, 2007). *ACS2*, *ACS4*, and *E8* were also directly regulated by RIN. The increase in fruit ethylene production is largely driven by the biosynthetic genes *ACS2*, *ACS4*, *ACO1*, and *E8* (Lincoln et al., 1987; Barry, 2007). This series of expression changes in key maturity genes will ultimately affect the process of fruit ripening.

Overall, our results suggest that PpSnRK1α interacts with SIRIN, increasing the expression of *RIN*, regulating the expression of downstream ripening-related genes and promoting the fruit ripening. However, the process of fruit ripening is a very complicated regulation network, in which SnRK1 and RIN may play a key role. Phosphorylation of RIN by SnRK1 may achieve a series of downstream regulations of maturation, but it is not known if it is also possible to directly regulate RIN-targeted TFs or activities of the key enzymes in the ethylene pathway. In addition, SnRK1 plays an active role in promoting plant photosynthesis and accumulation of plant sugars and other metabolites, and whether these are also reasons for early fruit maturation still require further verification.

## Author Contributions

FP and WY conceived and designed the experiments. WY, GW, and JL performed the experiments. GW and YX contributed reagents, materials, and analysis tools. WY and FP wrote the paper.

## Conflict of Interest Statement

The authors declare that the research was conducted in the absence of any commercial or financial relationships that could be construed as a potential conflict of interest.

## Supplementary Material

Figure S1 The expression levels of *CTR2* and *ETR1* genes in the WT and transgenic fruits during different fruit developmental stages.

## Abbreviations

ACS2: 1-aminocyclopropane-1-carboxylic acid synthase 2
ACS4: 1-aminocyclopropane-1-carboxylic acid synthase 4
ADPase: ADP-glucose pyrophosphorylase
AP2a: APETALA2a
CNR: colorless non-ripening
ChIP: chromatin immunoprecipitation
DTT: dithiothreitol
E8: ripening-associated ACO homolog
FUL1/TDR4: fruitfull 1
HEPES: 4-(2-Hydroxyethyl)-1-piperazine-ethanesulfonic acid
NOR: non-ripening
RIN: ripening inhibitor
SNF1: sucrose non-fermenting 1
SnRK1: sucrose non-fermenting-1-related protein kinase
SS: sucrose synthase
TF: transcription factor
WT: wild type
