# Supplementary figures and images for: Overexpression of PpSnRK1α in Tomato Promotes Fruit Ripening by Enhancing RIPENING INHIBITOR Regulation Pathway
Source: Front Plant Sci. 2018 Dec 17;9:1856. doi: 10.3389/fpls.2018.01856 (PMC6304366; doi:10.3389/fpls.2018.01856)

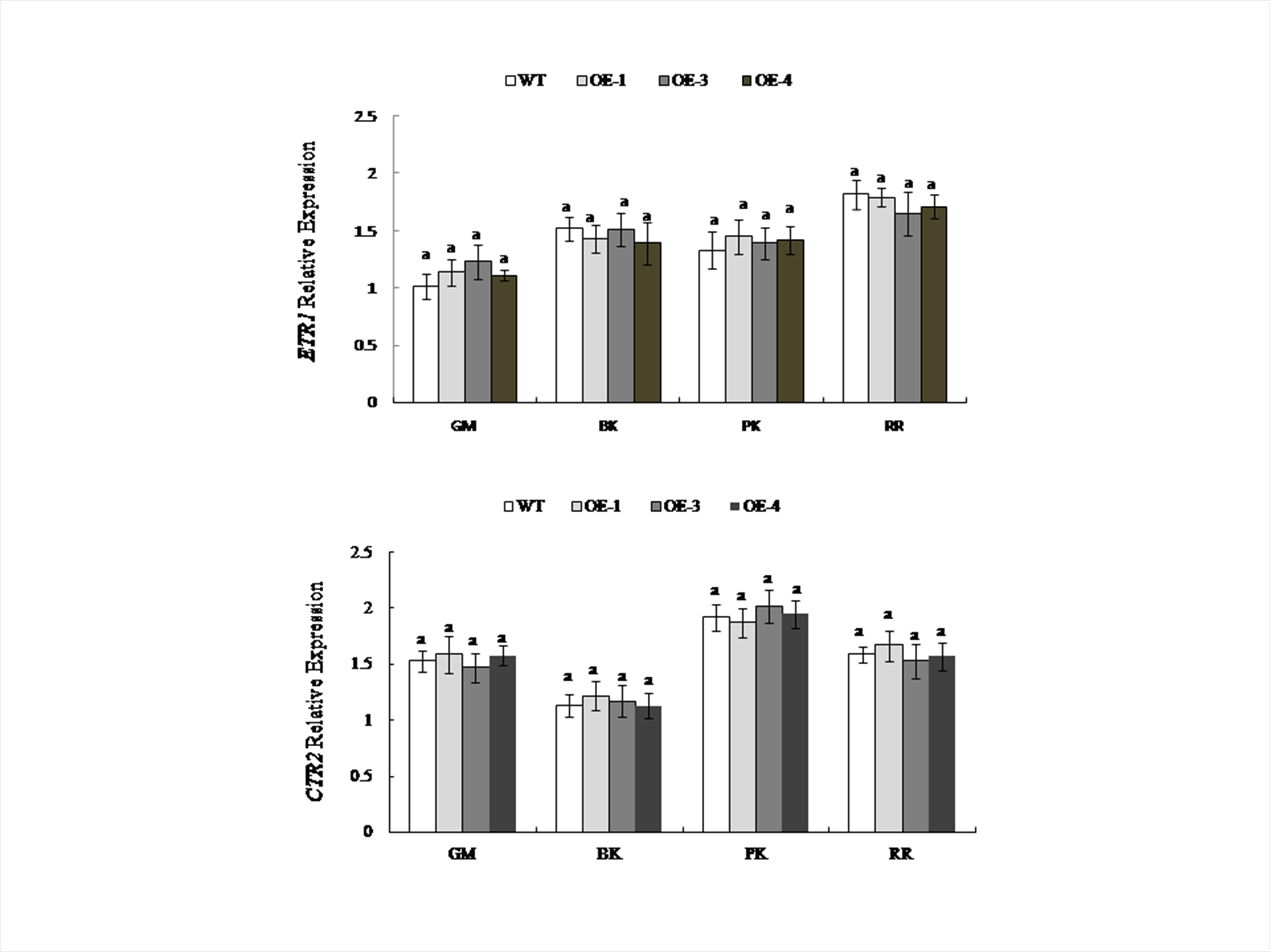

Supplement: Figure S1 — The expression levels of CTR2 and ETR1 genes in the WT and transgenic fruits during different fruit developmental stages. [file Image_1.TIF]
